# Level of Knowledge of Medical Staff on the Basis of the Survey in Terms of Risk Management, Associated with *Clostridioides difficile* Infections

**DOI:** 10.3390/ijerph18137060

**Published:** 2021-07-01

**Authors:** Zofia Maria Kiersnowska, Ewelina Lemiech-Mirowska, Katarzyna Semczuk, Michał Michałkiewicz, Aleksandra Sierocka, Michał Marczak

**Affiliations:** 1 Department of Management and Logistics in Healthcare, Medical University of Lodz, 90-419 Lodz, Poland; ewelina.l.mirowska@wihe.pl (E.L.-M.); adreslewska@wp.pl (A.S.); michal.marczak@umed.lodz.pl (M.M.); 2 Laboratory of Epidemiology, Military Institute of Hygiene and Epidemiology (WIHE), 01-163 Warsaw, Poland; 3 Department of Clinical Microbiology and Immunology, The Children’s Memorial Health Institute, 04-730 Warsaw, Poland; k.semczuk@ipczd.pl; 4 Faculty of Environmental Engineering and Energy, Institute of Environmental Engineering and Building Installations, Poznan University of Technology, 60-965 Poznan, Poland; michal.michalkiewicz@put.poznan.pl

**Keywords:** *Clostridioides difficile*, CDI, infection risk management, hand hygiene, medical staff

## Abstract

Infections caused by the toxigenic strains of *Clostridioides difficile* in the hospital environment pose a serious public health problem. The progressive increase in hospital infections in Poland indicates that risk management is a tool that is not used in an effective way and significantly differs from the goals set by the Leading Authorities, the Ministry of Health and its subordinate units. Systematic education of medical personnel constitutes the basic element of rational risk management aimed at reducing the number of infections as it allows for the transfer of knowledge, development of appropriate organizational procedures, and improves internal communication. This paper presents the results of a survey conducted in hospital facilities throughout Poland. The study dealt with what medical personnel know about channels of transmission and prevention of *Clostridioides difficile* infections in the hospital setting, professional training and risk management in terms of reducing the number of infections. The survey reveals that *Clostridioides difficile* continues to be a serious problem in the inpatient care system. Procedures and management strategies implemented by hospitals in order to limit the spread of the pathogen are predominantly focused on short-term action, which does not lead to a real improvement in terms of hospitalized patients’ safety. The infection risk management system was assessed at a fairly low level. The obtained research results confirmed the research hypotheses that had been formulated.

## 1. Introduction

Nosocomial infection of the *Clostridioides difficile* (*C. difficile*) etiology contributes, in most cases, to the prolonged hospitalization of a patient, whose underlying disease is not associated with the pathogen mentioned. Other important determinants increasing the risk of *C. difficile* infection (CDI) are the patient’s old age, the implementation of antibiotic therapy, invasive medical procedures, multi-patient rooms and direct contact with medical personnel, which may be the carrier of *C. difficile* spores, especially in a situation where hospital hygiene standards do not exist in practice [1,2,3,4]. It should be emphasized that hospitalization of the patient is nothing other than placing him or her in a potentially contaminated environment. The patient is exposed to viruses, microscopic fungi and bacteria [5,6,7]. Among nosocomial pathogens, *C. difficile* is the leading bacterium, which accounts for over 40% of all infections in inpatient treatment, followed by the following pathogenic microorganisms: *Acinetobacter baumannii* (8.56%), *Klebsiella pneumoniae* MBL (7.27%), Noroviruses (6.95%), Rotaviruses (6.62%) and *Staphylococcus aureus* MRSA (1.94%) [7]. According to the literature, the hands of medical personnel are the most common vector for the transmission of pathogens [8,9,10,11]. A study conducted by Otter et al. demonstrated that *C. difficile* spores were detected on the hands and gloves of medical personnel, who only spent some time with the patient diagnosed with CDI in the same room and had no direct contact with him through touch [12]. This proves a high level of contamination of rooms occupied by patients infected with the toxigenic strains of *Clostridioides*. Air movement occurring in the room causes dust and dust particles to float, together with biological agents such as spores, bacteria cells, fungal spores, etc., accompanied by the limitations of regular cleaning and disinfection of rooms with appropriately selected chemicals, increases the risk of transmission of infections.

Medical personnel play an important role in the diagnosis and treatment of the patient, and at the same time their presence is important in reducing or increasing the risks associated with the occurrence of hospital-acquired infections (HAI). Evidence suggests that the correct hand hygiene (HH) procedures reduce the frequency of infections caused by *C. difficile* [9,13]. Centers for Disease Control and Prevention (CDC) indicate that properly performed HH by medical workers is an effective and also the cheapest method of fighting the HAI epidemic. At the same time, it is also the basic action aimed at reducing the risk of transmission of *C. difficile* spores in medical facilities [14].

There are many underlying reasons behind failures of medical personnel to assess hand decontamination. One of them being the lack of awareness of possible consequences, insufficient knowledge about the microbiological factors present in the work environment (alarm pathogens), lack of knowledge about the routes of transmission of pathogens, bad habits or just plain unwillingness to comply with the imposed procedures [5]. At the core of this state of affairs is primarily shelving the process of continuous training and controlling employees, both in quantitative and qualitative terms, which adversely affects the behavior patterns and, as a consequence, contributes to the number of errors reported as undesirable events [5,15]. The professional experience of a team of medical professionals has a functional impact on the further professional conduct of junior medical personnel in terms of compliance with procedures and rules related to the patient’s safety.

The level of knowledge about the risk of infections caused by *C. difficile* among those with the least work experience is largely below the required level. It happens that young healthcare professionals, whose knowledge and practice in terms of risk of infection is still poorly grounded, are packed into the framework of the prevailing system and often do not receive appropriate professional support from more experienced employees. The literature demonstrates that the level of experience and knowledge varies among different groups of medical personnel. Nursing staff follow hygienic procedures much more frequently than other medical personnel [16,17,18]. Among the medical professions, doctors, and nurses in particular, represent a group that has the most frequent and direct contact with the patient. For this reason, they are expected to take an attitude of responsibility for the health and life of hospitalized patients, which at the same time translates into the health condition of the medical personnel themselves, as hygiene procedures equally serve the safety of medics performing their work in a potentially infectious environment. Year after year, medical personnel suffer from HAI. In Poland, per 678 outbreaks, on average 271 medical workers become infected in hospital epidemic outbreaks every year. The main microorganism responsible for causing nosocomial outbreaks is *C. difficile* (36.52%) [19].

An important risk factor behind CDI is deficiencies in the organization of the work of hospital units and hospital epidemiological supervision organizations, i.e., the total absence or inadequate operation of the hospital in the process of managing the risk of infections. As part of the measures to eliminate the identified risk, e.g., an epidemic outbreak, short-term solutions are often implemented at the stage of risk reduction. However, in the long term, those ad hoc solutions are not adequate to the epidemiological conditions prevailing in the ward and the behavior of medical personnel concerning their compliance with procedures. In addition, standard procedures implemented in the medical entity often do not directly correspond to the improvement of patient safety and the increase in the quality of medical services. Procedures are often introduced without extensive consultation with medical staff, without targeted training and without taking into account the current situation in the unit, which consequently leads to difficulties in the implementation of the basic tasks of the unit, conflicting messages, and finally to medical errors.

The infection risk management system in a hospital should be based on well-developed schemes and procedures which are known to be able to bring substantial benefits, for example through the maximum reduction of the risk to an acceptable level that would not pose a threat to the functioning of the unit [15].

The main objective of the study was to verify the following hypotheses:

- Medical personnel can be a vector for the transmission of infections caused by *C. difficile*;
- Medical workers’ knowledge of *C. difficile* is insufficient;
- Shortcomings related to the implementation of medical procedures are the result of a limited number of specialist training courses for medical personnel.

In the study, which was carried out in medical facilities throughout Poland, an attempt was made to verify the presented hypotheses on the basis of research surveys. In addition, the answers provided by the respondents to whom the questionnaire was addressed were used to check the following:

- what is the level of knowledge of medical personnel regarding the ways of spreading and preventing the transmission of infections caused by *C. difficile* in hospitalized patients?
- what is the current status of activities aimed at reducing HAI transmission?
- are the infection risk management tools used in an optimal and adequate manner to the threat level?
- does the seniority and professional experience of medical employees translate into the safety of medical services in terms of the frequency of adverse events?

## 2. Materials and Methods

This paper presents a study conducted in medical facilities throughout Poland. The research group consisted of medical personnel working in the hospital environment: nurses, doctors, paramedics, physiotherapists and other medical professionals employed in medical facilities and having direct contact with the hospitalized patient. The study implemented a diagnostic survey method with the use of the questionnaire technique. The survey was the author’s own research questionnaire. Participation in the study was voluntary and anonymous. In total, 1674 respondents took part in the survey. The first part of the questionnaire consisted of demographic questions concerning education, seniority, workplace and voivodship. The second part of the questionnaire consisted of 9 questions about the tools used in a hospital related to CDI risk management, based on the verification of the level of knowledge of medical personnel. The study dealt with the knowledge of medical personnel about the ways of spreading and preventing the transmission of infections caused by *C. difficile* in the hospital environment, professional training and risk management in terms of reducing the number of infections. The respondents could obtain a total of 0–10 points, where 0 points meant a lack of knowledge and 10 points full knowledge.

In the survey, which was addressed to a specific professional group, the following sociodemographic characteristics of the respondents were taken into account: medical profession, place of work (hospital treatment ward, hospital non-surgical ward, clinic), work experience (from 0–5 years to over 20 years with a 5-year limit) and voivodeship (approximate location of the workplace). No questions were asked about gender, age, income level and education background. This was due to the fact that at the end of 2020, only 2.5% of male nurses were registered in Poland, and this profession is dominated by women. In the case of doctors, this profession is more often practiced by women (58.4%), however, there is no available data about the gender of paramedics and other medical professions. Table 1 summarizes the available data on the occupational structure of selected healthcare providers in Poland.

In the data analysis, elements of descriptive statistics were used and the percentages of answers to the questions asked were calculated. The answers to the questions were described in terms of the number (*n*) and frequency (%). The level of knowledge was described using the basic statistical parameters: arithmetic mean ($\bar{x}$), standard deviation (Std. Dev., SD), median (M), lower and upper quartile (Q25 and Q75) and the minimum and maximum value (Min. and Max.). Non-parametric U Mann–Whitney and Kruskal–Wallis tests as well as Pearson chi-square, Yates corrected chi-square and NW chi-square (maximum likelihood) were used for statistical analyses. The following symbols were implemented in individual tests: Z—U Mann–Whitney test result; H—Kruskal–Wallis test result; p—probability level. In the statistical analysis, the *p* < 0.05 value was considered statistically significant. Statistical calculations were performed using the STATISTICA 10 PL statistical package.

## 3. Results

### 3.1. General Characteristics

In total, 1674 members of medical personnel participated in the study, of which the largest group, 1133 people (67.7%) consisted of nurses, then 306 (18.3%) doctors and 235 (14%) medical staff who were paramedics, physiotherapists working in the hospital at treatment and non-surgical wards and other medical professionals employed in medical facilities and having direct contact with the hospitalized patient. In the study, an overwhelming majority of workers were employed in hospitals in surgical and non-surgical departments (81.4%), and the rest of the medical staff were employees of medical clinics (18.6%). The most active group participating in the questionnaire was medical personnel from Masovian Voivodeship (293 people), Silesian (211 people) and Lower Silesia (146 people), where nurses constituted 17.2%, 14.6% and 8.0%, respectively, and other medical professions accounted for 18.1%, 8.5% and 10.2%, respectively (doctors, paramedics, hospital physiotherapists, and other medical professionals employed in a hospital).

When analyzing seniority, the highest percentage among all medical personnel were people with over 20 years of experience (33.4%), followed by those with the shortest work experience: 0–5 years (32.5%).

There was a clear difference in terms of seniority among the medical professions. Nurses working in the profession for over 20 years (45.1%) and those working in the range of 0–5 years (24.4%) prevailed, while the other medical professions were dominated by doctors with the shortest seniority of 0–5 years (49.4%) and 5–10 years (23.9%). The above data was statistically significant (*p* < 0.0001).

The analysis of responses to individual questions assembled on the basis of a survey.

### 3.2. Knowledge of Healthcare Professionals on Hand Hygiene Procedures and Infections Caused by C. difficile

- Self-assessment of knowledge about the hand hygiene procedure: (*p* = 0.0002)—a very good and good score was given by 93.4% of all medical personnel, while average and insufficient score was provided by 6.6%. Better knowledge was demonstrated among the nurses and the worse among the rest of the medical staff (doctors, paramedics working in the hospital and other medical professionals employed in the hospital).
- Self-assessment concerning knowledge about CDI: (*p* < 0.0001)—it was graded as very good and good by 70.1% of all medical personnel, whereas as average or insufficient by 29.9%. Higher percentages of worse results were given by the remaining medical personnel.
- Awareness of the ways CDI are spread in the hospital setting: (*p* < 0.0001)—the majority of medical personnel knew the routes of transmission of CDI (77.1%), however 22.9% of the respondents lacked this knowledge.
- How often hand hygiene procedures are followed at work: (*p* = 0.0120)—in 93.8% of cases those procedures are performed routinely, and in 6.2% of cases they are performed rarely or sometimes even totally forgotten.
- The number of professional trainings in hand hygiene procedures organized by the employer: (*p* = 0.7540)—according to 52.1% of the respondents, it is sufficient or too frequent, while as many as 47.9% claim that it is too small. Very similar percentages were reported among nurses and other medical personnel.
- The frequency of supervision for the implementation of the hand hygiene procedure in the workplace: (*p* < 0.0001)—the vast majority, i.e., 44.6%, reported very rare controls, while controls that happen every quarter, every six months and once a year accounted for 19.3%, 17.5% and 18.6% respectively.
- In cases where the employer carried out periodic inspections of the compliance with the hand hygiene procedure: the most common (50.1%) was a check-up performed by an epidemiologist or an epidemiological nurse (*p* = 0.0059), but according to the second common answer (26.9%), no controls were performed (*p* < 0.0001).

### 3.3. Answers to Questions Checking the Knowledge of Medical Personnel

- To the question: Does proper hand hygiene of medical personnel influence nosocomial infections? (*p* = 0.0017)—99.0% of employees gave the correct answer and 1.0% chose the wrong answer.
- To the question: Purposefulness of the hand hygiene procedure: (*p* = 0.0014)—91.8% of the respondents gave the correct answer and 8.2% responded wrongly.
- To the multiple-choice question: (*p* = 0.0002)—in a hospital, cross-infection may happen via the following: the most responses indicated that by transmission of microorganisms from an infected patient to another patient (85.9%) and from an infected patient to medical personnel (77.6%). To the question: The main vector of transmission of nosocomial infections is: (*p* < 0.0001)—the majority of the respondents (93.7%) indicated hands of medical personnel, whereas 6.4% of them pointed to reusable and disposable equipment.
- To the question: When should a hand hygiene procedure be performed when it in necessary to examine a patient suspected of having CDI: 95.3% of the respondents gave the correct answer that both before and after the examination of the patient, while 4.7% submitted a wrong answer.
- To the question: Please choose a hand hygiene method when medical staff have contact with a patient infected or suspected of having CDI: (*p* < 0.0001)—the correct answer (washing hands under running water with soap) was given by 59.0% of the employees, but as much as 41.0% of the respondents answered wrongly to this question.
- To the question: According to the procedure, the time spent on hand hygiene when staff has contact with a patient infected or suspected of being infected with *C. difficile* should take: the correct answer (40–60 s) was given by 53.5% of the medical personnel, while as much as 46.5% of the respondents chose too short hand washing time (for *p* = 0.0065).
- To the question: What is your opinion on the hand hygiene procedure: (*p* = 0.0401)—the correct answer (protects against infection transmission) was submitted by 98.6% of the respondents, and 1.4% gave the wrong answer.
- To the question: “bare below the elbows” policy (BBE—this strategy involves the dress code of medical personnel. It consists of eliminating wearing jewelry and ties by medical employees, additionally wearing short-sleeved aprons in winter, as well as eliminating wearing varnished and long nails): (*p* < 0.0001)—the correct answer (limiting the patient’s contact with contaminated clothing of medical personnel, promoting hand and wrist hygiene) was given by 40.3% of the respondents, while 59.7% gave incorrect answers.

### 3.4. Level of Knowledge of Medical Personnel

The level of knowledge of medical personnel was determined on the basis of the sum of points obtained for correctly answered questions. Table 2 summarizes the descriptive statistics of the level of knowledge for all medical workers, nurses and other medical personnel (doctors, paramedics working in a hospital and other medical professionals employed in a hospital).

**Figure 1:**
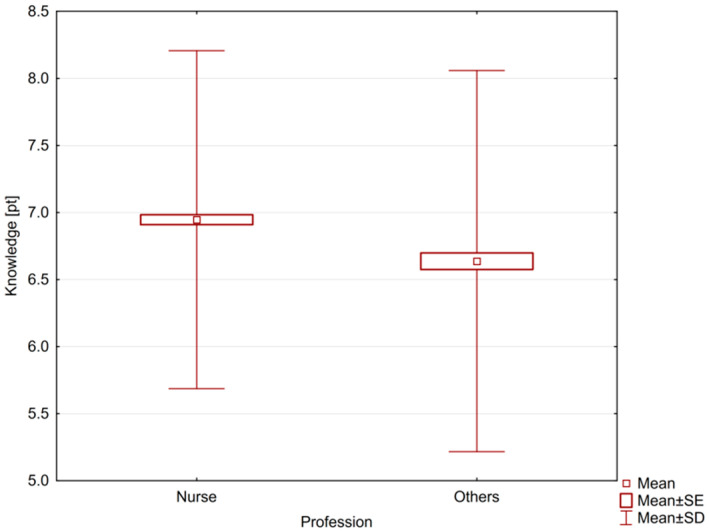
The level of knowledge of nurses and other medical workers.

A *p* < 0.05 value was considered statistically significant. The average knowledge of all medical personnel was 6.85 ± 1.32, in the case of nurses it was more profound than in the case of other employees—6.95 ± 1.26 and 6.64 ± 1.42, respectively. This difference was statistically significant (*p* = 0.0001). Nurses were characterized by greater knowledge than other medical personnel (Figure 1).

### 3.5. Analysis of the Workplace and the Frequency of Monitoring the Implementation of Hand Hygiene Procedures

In the questionnaire, the workplace of the medical staff was divided into the following categories: a hospital treatment ward, a non-surgical ward and a clinic. Table 3 shows the relationship between the workplace and the examination on the implementation of the hand hygiene procedure in the workplace of medical personnel.

There is a statistically significant relationship between the workplace and the control frequency of the hand hygiene procedure in the workplace of medical personnel (*p* < 0.0001).

Quarterly checkups were most frequent among respondents working in a hospital surgical ward (21.8%) or in a non-surgical hospital ward (21.0%). Once a year inspections were most common among respondents working in a hospital treatment ward (20.9%), and very rare inspections were most often carried out among respondents working in an outpatient clinic (60.8%).

**Figure 2:**
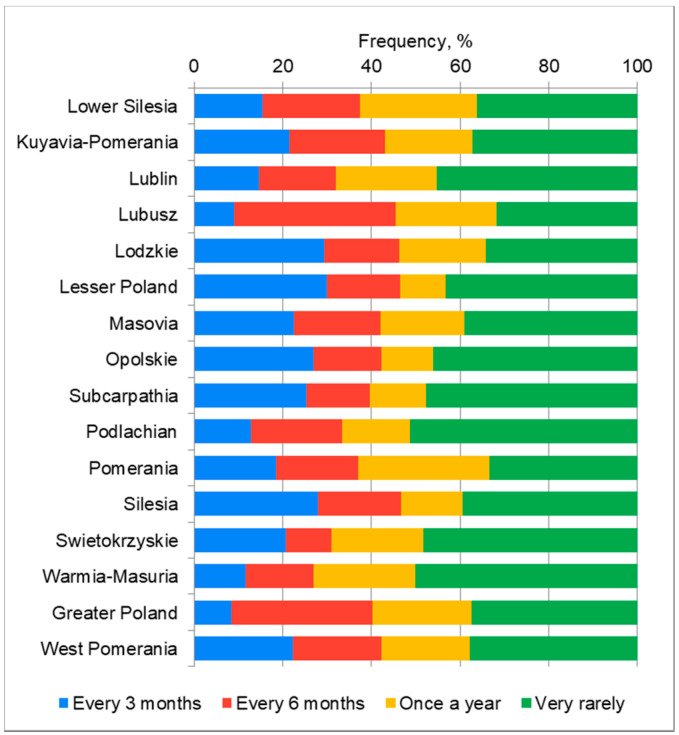
The frequency of inspections of the implementation of the hand hygiene procedure in the workplace in individual voivodships.

There is a statistically significant correlation between the voivodship and the frequency of the hand hygiene procedure controls in the workplace (*p* = 0.0082). Most often, the respondents indicated very rare checks on the implementation of the hand hygiene procedure (Figure 2).

### 3.6. Analysis of the Workplace and the Tools Used by the Employer When Conducting Periodic Inspections of Medical Personnel

On the basis of statistical calculations and the results of the Pearson chi-square test, the relationship between the workplace and tools used by the employer when carrying out periodic checks on hand hygiene procedure compliance, it was found out that supervision performed by an epidemiologist/epidemiological nurse in non-surgical (54.2%) and surgical hospital wards (54.1%) was the most frequently used method. On the other hand, in most clinics (49.8%) checks were not carried out at all.

### 3.7. Assessing Various Factors That Have an Impact on Giving the Correct Answer

#### 3.7.1. Work Experience in the Profession vs. the Correct Answers to the Knowledge Check Questions

Statistically significant differences between the respondents with different seniority in the profession were found for the following questions:

- Hand hygiene procedure: (*p* = 0.0084)—the correct answer was most often given by respondents with over 20 years of work experience (94.3%) and 10–15 years of work experience (94.1%).
- The main vector of transmission of nosocomial infections is: (*p* < 0.0001)—the correct answer was most often given by respondents with over 20 years of work experience (97.0%) and 15–20 years of work experience (96.8%).
- Please select a hand hygiene method when staff come into contact with a patient that is either infected or is suspected of being infected with *C. difficile*: (*p* = 0.0102)—the correct answer was most often provided by respondents with work experience up to 5 years (64.5%).
- According to the procedure, the time spent on hand hygiene when staff come into contact with a patient that is either infected or suspected of having CDI should take: (*p* <0.0001)—the correct answer was most often provided by respondents with up to 5 years of work experience (62.1%).
- “Bare below the elbows” policy (BBE) concerns: (*p* < 0.0001)—the correct answer was most often given by respondents with up to 5 years of work experience (51.6%).

#### 3.7.2. Workplace vs. the Correctly Answered Knowledge Check Questions

Statistically significant differences between the respondents from different workplaces were found for the following questions:

- Hand hygiene procedure: (*p* = 0.0016)—the correct answer was most often provided by respondents working in hospital surgical wards (93.3%) and non-surgical wards (92.6%).
- The main vector of transmission of nosocomial infections is: (*p* = 0.0146)—the correct answer was most often provided by the respondents working in non-surgical (95.3%) and surgical (93.7%) wards.
- Please select a hand hygiene method when staff come into contact with a patient that is either infected or is suspected of being infected with *C. difficile*: (*p* = 0.0003)—the correct answer was most often provided by the respondents working in a hospital ward (61.7%) and non-surgery ward (60.8%).

#### 3.7.3. Self-Assessment of Knowledge about the Hand Hygiene Procedure vs. Correctly Answered Knowledge Check Questions

Statistically significant differences between the respondents with different self-assessment of knowledge about the hand hygiene procedure were found for the following questions:

- The main vector of transmission of nosocomial infections is: (*p* = 0.0134)—the correct answer was most often given by the respondents who described their knowledge about hand hygiene as good (94.5%) and very good (93.6%).
- Please choose a hand hygiene method when staff come into contact with a patient that is either infected or is suspected of being infected with *C. difficile*: (*p* = 0.0420)—the correct answer was most often provided by the respondents who described their knowledge about hand hygiene as good (61.9%).
- “Bare below the elbows” policy (BBE) concerns: (*p* = 0.0102)—the correct answer was most often given by the respondents who described their knowledge about hand hygiene as good (43.9%).

#### 3.7.4. Self-Assessment of Knowledge about CDI vs. Correctly Answered Knowledge Check Questions

Statistically significant differences between respondents with different self-assessments of knowledge about CDI were found for the questions:

- Is the number of nosocomial infections influenced by the proper hand hygiene performed by healthcare professionals? (*p* = 0.0332)—the correct answer was most often given by the respondents who described their knowledge about CDI as good (99.5%), average (99.1%) and very good (98.3%).
- The main vector of transmission of nosocomial infections is: (*p* = 0.0006)—the correct answer was most often given by the respondents who described their knowledge about infections caused by *C. difficile* as good (95.4%) and very good (93.6%).
- A medical worker is to examine a patient suspected of having CDI, in which case the procedure involving hand hygiene should be carried out: (*p* = 0.0025)—the correct answer was most often given by the respondents who described their knowledge as average (96.4%), good (95.6%) and very good (94.8%).
- Please choose a hand hygiene method when staff come into contact with a patient that is either infected or is suspected of being infected with *C. difficile*: (*p* < 0.0001)—the correct answer was most often provided by the respondents who described their knowledge about CDI as very good (67.0%).
- According to the procedure, the time spent on hand hygiene when staff come into contact with a patient that is either infected or suspected of having CDI should take: (*p* = 0.0019)—the correct answer was most often given by the respondents who described their knowledge about CDI as very good (57.1%) and average (57.0%).
- What is your opinion on the hand hygiene procedure? (*p* = 0.0006)—the correct answer was most often provided by the respondents who described their knowledge about CDI as average (99.3%), good (98.9%) and very good (98.3%).

#### 3.7.5. Number of Professional Trainings vs. Correctly Answered Knowledge Check Questions

Statistically significant differences between the respondents with different opinions on the number of professional trainings on hand hygiene organized by the employer were found for the following questions:

- Is the number of nosocomial infections influenced by the proper hand hygiene of healthcare professionals? (*p* = 0.0058)—the correct answer was most often given by the respondents who believed that the number of trainings was too small (99.3%) and sufficient (99.2%).
- The main vector of transmission of nosocomial infections is: (*p* = 0.0038)—the correct answer was most often given by the respondents who believed that the number of trainings was sufficient (94.1%) or too small (93.9%).
- A medical worker is to examine a patient suspected of having CDI, in which case the procedure involving hand hygiene should be carried out: (*p* = 0.0003)—the correct answer was most often provided by the respondents who believed that the number of trainings was sufficient (96.2%) or too small (95.0%).
- What is your opinion on the hand hygiene procedure? (*p* < 0.0001)—the correct answer was most often given by the respondents who believed that the number of trainings was sufficient (99.2%) or too small (98.9%).

#### 3.7.6. The Frequency of Controls on the Implementation of the Hand Hygiene Procedure in the Workplace vs. Correctly Answered Knowledge Check Questions

A statistically significant difference between the respondents having different frequency of checks on the implementation of the hand hygiene procedure was found only for the question: “Bare below the elbows” policy (BBE) concerns: (*p* = 0.0377)—the correct answers were most often provided by the respondents who experienced checks very rarely (43.8%).

### 3.8. Analysis of Work Experience in the Profession and the Level of Knowledge among Medical Personnel

Table 4 summarizes descriptive statistics of the respondents’ knowledge depending on the seniority/length of service.

The Kruskal–Wallis test showed a statistically significant difference in the level of knowledge between respondents with different lengths of service in the profession (*p* = 0.0005).

On the basis of additional calculations of the probability level of the *p* multiple comparison test, it was found that significant statistical differences in the level of knowledge occurred only between 0–5 years and 5–10 years (*p* = 0.0129) and between 0–5 years and over 20 years (*p* = 0.0015). In both cases, the average of knowledge was higher in the respondents working for no more than 5 years.

### 3.9. Self-Assessment of Knowledge on the Hand Hygiene Procedure vs. the Level of Knowledge among Medical Personnel

Table 5 presents descriptive statistics of the level of knowledge on hand hygiene.

The Kruskal–Wallis test showed a statistically significant difference in the level of knowledge between the respondents that self-evaluated their knowledge on the hand hygiene procedure (*p* = 0.0089). Only 6.6% of the respondents rated their knowledge as average or unsatisfactory.

### 3.10. Self-Evaluation of Knowledge on CDI vs. the Level of Knowledge among Medical Personnel

Descriptive statistics on CDI are summarized in Table 6.

The Kruskal–Wallis test showed a statistically significant difference in the level of knowledge between respondents that self-evaluated their knowledge on CDI in a different way (*p* < 0.0001). Among those who participated in the survey, 29.8% of the respondents had average or insufficient knowledge.

### 3.11. Performing Hand Hygiene Procedures vs. the Level of Knowledge among Medical Personnel

Table 7 shows descriptive statistics on the level of knowledge and frequency of performing the hand hygiene procedure.

The Mann–Whitney U test showed a statistically significant difference in the level of knowledge between the respondents who perform the hand hygiene procedure with different frequency (*p* = 0.0465). Subjects who routinely performed hand hygiene procedures demonstrated on average greater knowledge than the rest, while 6.2% of the respondents demonstrated smaller knowledge and had a tendency to forget about hand hygiene procedures or to perform them rarely.

### 3.12. The Number of Professional Trainings vs. the Level of Knowledge among Medical Personnel

Table 8 shows descriptive statistics on the level of knowledge and opinions given on the number of trainings.

The Kruskal–Wallis test showed a statistically significant difference in the level of knowledge between respondents with different opinions on the number of trainings organized by the employer in the field of hand hygiene procedures (*p* = 0.0123). Despite a fairly positive average rating, as many as 47.9% of the respondents believe that the number of trainings is too small.

### 3.13. The Frequency of Checks on the Implementation of the Hand Hygiene Procedure in the Workplace vs. the Level of Knowledge among Medical Personnel

Table 9 shows descriptive statistics on the level of knowledge and varying frequency of hand hygiene controls.

The Kruskal–Wallis test did not show a statistically significant difference in the level of knowledge among the respondents with varying frequency of checks on the hand hygiene procedure implementation (*p* > 0.05). The frequency of controls on the hand hygiene procedure did not affect the level of knowledge among the respondents.

After evaluating the survey results, it can be stated that answers to the posed questions were obtained and that research hypotheses were confirmed:

- Despite the average score of 6.85 (out of 10 possible) obtained for correctly answered questions that evaluated the knowledge of medical workers, this value is not satisfactory as differences in the level of knowledge among different groups were noticed. The average rating obtained by the nurses was higher (6.95) than the average score achieved by the other medical workers (6.64).
- The knowledge of healthcare professionals about *C. difficile* and the transmissions of CDI in a hospital environment is unsatisfactory.
- The risk management of infections in hospitals that included, among others, training and control of the spread of *C. difficile* outbreaks, was assessed to be at a fairly low level. The number of professional training courses for medical personnel is insufficient to meet the demand, and hand hygiene supervision does not in fact happen in practice. This is also due to insufficient understanding of the staff about ways of transmission of infections, poor knowledge of methods of approaching the patient while still maintaining a satisfactory level of hand hygiene.
- Seniority and professional experience play an important role in preventing HAI. Most of the questions relating to general hospital hygiene were best answered by people with longer work experience, like 20 years or more, and also by those with 10–15 years of work experience. On the other hand, questions about the ways of spreading and preventing transmission of infections caused by *C. difficile* were better answered by people with short work experience, i.e., ranging from 0 to 5 years.
- The survey analysis showed that transmission of *C. difficile*-related infections may be a difficult problem to solve in hospitals. This is confirmed not only by the unsatisfactory results of medical personnel’s knowledge about *C. difficile* and the ways of spreading this pathogen, but also by annual epidemiological data (NIH, GIS), which indicate that the number of CDI continues to grow. In 2019, there was 9698 cases, compared to 4457 in 2013. On the top of that, from among all alarm factors causing outbreaks in hospitals in 2019, *C. difficile* accounted for the largest percentage, namely 33%.

## 4. Discussion

In recent years, many publications on *C. difficile* [23,24,25,26,27,28,29,30,31,32,33,34,35] have been published, but the vast majority of them presented general information about the microorganisms, the number of diagnosed cases, methods of diagnosis, patient treatment and transmission routes. Definitely much fewer papers discuss the issues related to the knowledge of healthcare professionals about *C. difficile*, seniority, the etiology of CDI in medical facilities, costs of treating patients with nosocomial infections and the responsibility of hospital management for training and supervising subordinate medical staff.

What constitutes an important element in the aspect of reducing the spread of respiratory infections caused by *C. difficile* is the knowledge about the real existence of the threat posed by the development of CDI in hospitalized patients, understanding and observance of basic hygiene rules, as well as following sanitary regime in detected outbreaks. Medical unit managers, Hospital Infection Control Teams and healthcare professionals are forced to mutually control the level of safety in the field of provided medical services and to constantly improve their skills through professional development. It is extremely important in the functioning of a medical facility that employees are aware of the fact that sometimes seemingly trivial violations of procedures or hygiene rules may have serious consequences in the form of an epidemic, which, in turn, may lead to the complete closure of a department or part of a unit and thus disrupt a normal medical activity.

In our studies conducted throughout Poland, it was found that the awareness of CDI among healthcare staff is insufficient. Nurses demonstrated a higher level of knowledge than other medical workers. Additionally, among the respondents it was found that as many as 22.9% did not have any understanding about CDI routes of transmission in the hospital setting. The knowledge about the ways of spreading and preventing the transmission of infections varies depending on the country. In Nigeria, in the largest tertiary hospital, the knowledge of medical staff was classified as below adequate [17]. The study by Burnett and Kearney has demonstrated a low level of knowledge and risk perceptions of *C. difficile* among medical staff [36]. In contrast, it was proven in a reference teaching-research hospital in northern Italy that time spent on acquiring knowledge about CDI can have a substantial impact on a positive attitude towards established procedures, including HH. It was found that the lower number of professional trainings for nurses translated not only into an increase in CDI by 36%, but also into a higher number of in-hospital mortality cases. It was also concluded that continuous hospital workers education could be more important than the possibility of isolating infected patients by putting them in single rooms [37]. That study demonstrated that the Hospital Infection Control Team correctly identified the relationship of intensified education and training in CDI cases and related mortality among patients when analyzing the CDI risk assessment. As part of the infection risk management process, at the stage of analyzing possible solutions, no decision was made to accept the risk, but on the contrary, risk minimization measures were implemented, additionally taking into account the costs of implementing CDI-related anti-epidemic measures. Another and important factor in risk assessment, apart from infection monitoring, is the control of medical processes such as the proper HH performed by hospital workers.

The awareness of healthcare staff and the ability to apply appropriate procedures play an essential role in minimizing the risk of infections. Our study showed a significant difference in the level of knowledge among healthcare professionals and the actual frequency and quality of HH procedure performance. The average knowledge was greater among those who routinely performed the HH procedure, while 6.2% of the respondents demonstrated less knowledge and they had the tendency to perform HH procedures rarely or even totally forgot about them. This is also confirmed by numerous studies that healthcare staff not only should possess appropriate knowledge, but also should be adequately prepared, both theoretically and practically, to properly perform professional tasks. The study conducted by Ramadan and Hamz, like our own study, demonstrated that the frequency of performing HH procedures was directly proportional to the level of awareness and knowledge among medical staff, i.e., after a higher and targeted number of professional trainings [38]. In addition, our study revealed that as many as 41% of healthcare professionals did not know how the HH procedure should be adequately performed in the case of having contact with a patient infected or suspected of having CDI, whereas 4.7% of the respondents did not even know when a given procedure should be performed. This result shows that intensified training and feedback, as well as checks are necessary to maintain a high level of HH compliance [5].

The results of our study revealed differences in the state of knowledge among different groups of healthcare workers. The mean score obtained by the nurses (6.95 out of 10.0 possible) was higher than the average score obtained by the rest of the medical staff (6.64), which confirms that nurses are more compliant to HH-related procedures than doctors. A study by Omiye and Afolaranmi demonstrated that nurses were more likely to follow HH procedures (33.2%) when compared to doctors (29%) [18]. This might be because nursing students are taught about the HH procedures already at the stage of practical vocational training, whereas medical students are informed about the HH procedures only at a later stage of education and they tend to pay less attention to these aspects [39]. The existence of such a phenomenon demonstrates that a conscious approach to reducing the risk of HAI, including *C. difficile* infections, should be implemented and monitored at an early stage of professional practice.

Efficient minimization of infections in the risk management process, at the stage of monitoring the effects of implemented activities, is crucial. Monitoring these processes most often takes place after the implementation of risk reduction measures. In the context of infections, it is an answer to the question of whether the expected effect has been achieved as a result of the implementation of additional procedures, educational and research initiatives, while it is carried out through the verification of infection cases and supervision over the implementation of medical processes. Checks, on the other hand, may take place as part of an audit ordered by the employer and epidemiological controls carried out by the Hospital Infection Control Team. Risk management control is the process of systematic quantitative comparison of practice with new and current standards of conduct. This process informs, for example, about an increase or decrease in the number of infections and in the level of compliance with the procedures. In our study we obtained from the respondents some disturbing responses to the questions about supervision. Almost half of the respondents argue that epidemic checks are carried out very rarely or they do not remember when they were last ordered. The situation is similar with the number of inspections carried out by the employer. Epidemiological inspections were most often conducted in the workplaces of the respondents working in hospital treatment wards, very rarely in outpatient clinics. Numerous studies, as well as our own, confirm the strong correlation between the incidence of nosocomial infections and the number of professional trainings and controls, as well as the level of knowledge and frequency of properly performed medical procedures [5,18,37]. An Australian study by Grayson and Stewardson found that after 8 years of targeted training and intensive epidemiological checks implemented in 105 hospitals, the number of HAI had dropped by 15%. In this study, epidemiological checks on the compliance with the procedures and numerous trainings were significantly related to the obtained decrease in the number of nosocomial infections [40].

Evaluating the obtained results of the survey against literature data, it can be stated that the problem of infections caused by *C. difficile* exists not only in Poland. Knowledge about the routes of infection transmission, compliance with the principles of *C. difficile* transfer prevention, and the appropriate number of training courses and inspection controls are the basic tasks for both medical workers and control and management staff working in medical facilities. Considering the wide variety found among medical workers who have direct contact with the patient, efforts should be made to broaden and consolidate the knowledge about infections caused by *C. difficile*, regardless of whether it concerns doctors, nurses or other medical professionals, and regardless of the length of the seniority. It turns out that the theoretically good self-esteem of medical workers about their knowledge on *C. difficile* is not reflected in practice, and the number of infections both in Poland and in the world does not decrease but increases every year.

## 5. Conclusions

In medical facilities throughout Poland, infection risk management is based mainly on the passive observation of adverse events without conducting reliable analyses, providing feedback and active actions. The results of the study show serious gaps in the proper management of infection risk. Controls, compliance with the HH procedure, knowledge and awareness of the risk about the routes of transmission of CDI is unsatisfactory. This was reflected not only in our study, but also in annual epidemiological reports in Poland and around the world, which inform that the number of CDI is constantly growing.

The transmission of infections between medical staff and patients, and vice versa, between patients and medical staff, can be effectively limited, but it requires strict adherence to basic hygiene rules and appropriate medical procedures at the hospital. Proper rules of hospital hygiene, including the rules of washing hands described in the guidelines of the World Health Organization (WHO) [41], regular training in this field, hand cleanliness checks, rules and procedures for contacting a hospitalized patient are the main tasks that should function in every hospital. They apply to nurses, doctors and other medical workers.

The management of the medical facility and managers of individual organizational units are responsible for their introduction, compliance and implementation. The sooner, more accurately and effectively these rules are introduced and enforced, the greater the chance of reducing the number of nosocomial infections and reducing hospitalization length. It should be emphasized that each prolonged stay of the patient in the treatment facility translates into an increase in the risk associated with the occurrence of an adverse event and is associated with specific costs that, in the aggregate, affect the negative financial balance of the medical entity.

CDI-related procedures must be kept up-to-date due to the severity of the problem. It is also necessary to develop internal control tools for the correct implementation of procedures related to the HH principles and for dealing with suspicion and/or confirmation of CDI. Each reported outbreak caused by *C. difficile* should be thoroughly analyzed by the Hospital Infections Team in terms of cause and effect, and the results and conclusions of the epidemiological investigation should be presented in an accessible and understandable manner to the ward staff in order to avoid similar mistakes in the future. Additionally, a more targeted number of screening tests on admission and microbiological diagnostics during hospitalization should be implemented, allowing for quick identification of patients colonized by *C. difficile* and taking preventive measures, i.e., implementing selective isolation of high-risk patients on admission to the hospital or isolation/cohortation of patients in the outbreak [42]. On a macro scale, this procedure is quite difficult and costly, as there are usually several patients in the rooms, but when analyzing the costs of the patient’s extended stay in the hospital and additional CDI treatment, it seems to be a solution that meets the needs related to increasing the level of safety in the process of patient hospitalization.

## Figures and Tables

**Table 1:** Classification structure of selected healthcare providers in Poland.

Medical Profession	Female	Male	Age up to 40 Years	Age +41
Nurse ^(1)^	295,571	7640	15%	85%
Doctor ^(2)^	82,892	59,138	26.8% of men and 32.7% of women	73.2% of men and 67.3% of women
Paramedic ^(3)^	10,300		No data	No data
Another medical profession	No data			

^(1)^ as of 31/12/2020, data from the Chamber of Nurses and Midwives [20]; ^(2)^ as of 31/05/2021, data from the Chamber of Physicians and Dentists [21]; ^(3)^ as of 31/12/2019, data from Statistics Poland without gender breakdown [22].

**Table 2:** Descriptive statistics of the respondents’ knowledge level by occupation and the Mann–Whitney U-test results (Z).

Parameter	Total *n* = 1674	Nurses *n* = 1133	Other Medical Personnel *n* = 541
Mean ($\bar{x}$)	6.85	6.95	6.64
Std. Dev. (SD)	1.32	1.26	1.42
Median (M)	7.0	7.0	7.0
Q25	6.0	6.0	6.0
Q75	8.0	8.0	8.0
Min.	1	2	1
Max.	10	10	10
Range	0–10	0–10	0–10
Z		3.83	
P		0.0001	

**Table 3:** Number (*n*) and frequency (%) of the respondents from different workplaces according to the given answers to the question: How often are hand hygiene controls performed in your workplace and the Pearson chi-square test result.

How Often Are Hand Hygiene Controls Applied in Your Workplace	Workplace						*p*
	Clinic *n* = 311		Hospital Treatment Ward *n* = 728		Hospital Non-Surgical Ward *n* = 633		
	*n*	%	*n*	%	*n*	%	
Every quarter	31	10.0	159	21.8	133	21.0	<0.0001
Every 6 months	43	13.8	137	18.8	113	17.9	
Once a year	48	15.4	152	20.9	111	17.5	
Very rarely; I don’t remember when	189	60.8	280	38.5	276	43.6	

A *p* < 0.05 value was considered statistically significant.

**Table 4:** Descriptive statistics of the level of knowledge among respondents with various lengths of work in the profession and the result of the Kruskal–Wallis test (H).

Seniority in the Profession	*n*	Knowledge (Points)					H	*p*
		Mean ($\bar{x}$)	Std. Dev. (SD)	Median (M)	Min.	Max.		
0–5 years	543	7.03	1.35	7.0	2	10	20.13	0.0005
5–10 years	264	6.73	1.38	7.0	2	10		
10–15 years	152	6.84	1.40	7.0	1	10		
15–20 years	154	6.71	1.31	7.0	2	9		
Over 20 years	559	6.76	1.23	7.0	1	10		

**Table 5:** Descriptive statistics of the level of knowledge in respondents with different self-esteem concerning their understanding of the hand hygiene procedure and the Kruskal–Wallis test results (H).

I Would Describe My Knowledge on the Hand Hygiene Procedure as:	*n*	Knowledge (Points)					H	*p*
		Mean ($\bar{x}$)	Std. Dev. (SD)	Median (M)	Min.	Max.		
Very good	738	6.74	1.30	7.0	1	10	9.44	0.0089
Good	824	6.96	1.31	7.0	2	10		
Average or insufficient	110	6.67	1.52	7.0	1	9		

**Table 6:** Descriptive statistics on the knowledge level of the respondents with different self-evaluation of their knowledge on CDI and the Kruskal–Wallis test results (H).

I Would Describe My Knowledge on CDI as:	*n*	Knowledge (Points)						
		Mean ($\bar{x}$)	Std. Dev. (SD)	Median (M)	Min.	Max.	H	*p*
Very good	233	6.85	1.32	7.0	1	10	23.61	<0.0001
Good	940	6.89	1.30	7.0	2	10		
Average	442	6.89	1.28	7.0	2	10		
Insufficient	57	5.82	1.59	6.0	1	9		

**Table 7:** Descriptive statistics on the level of knowledge among subjects performing the hand hygiene procedure with different frequency and the Mann–Whitney U test results (Z).

How Often do You Perform a Hand Hygiene Procedure at Work?	*n*	Knowledge (Points)						
		Mean ($\bar{x}$)	Std. Dev. (SD)	Median (M)	Min.	Max.	Z	*p*
Routinely	1568	6.87	1.28	7.0	2	10	1.99	0.0465
Rarely or I happen to forget	104	6.43	1.74	7.0	1	10		

**Table 8:** Descriptive statistics on the level of knowledge among respondents with different opinions on the number of hand hygiene trainings organized by the employer and the Kruskal–Wallis test results (H).

Do You Think That the Number of Hand Hygiene Trainings Organized by the Employer Is:	*n*	Knowledge (Points)						
		Mean ($\bar{x}$)	Std. Dev. (SD)	Median (M)	Min.	Max.	H	*p*
Too frequent	26	5.73	2.36	6.0	1	10	8.80	0.0123
Enough	845	6.88	1.28	7.0	3	10		
Too small	801	6.85	1.31	7.0	1	10		

**Table 9:** Descriptive statistics on the level of knowledge among the respondents with varying frequency of controls on the hand hygiene procedure implementation and the Kruskal–Wallis test results (H).

How Often Are Hand Hygiene Controls Organized in Your Workplace:	*n*	Knowledge (Points)						
		Mean ($\bar{x}$)	Std. Dev. (SD)	Median (M)	Min.	Max.	H	*p*
Every quarter	323	6.74	1.32	7.0	3	10	2.37	0.4996
Every 6 months	293	6.87	1.29	7.0	4	10		
Once a year	311	6.90	1.23	7.0	2	10		
Very rarely. I don’t remember when	745	6.86	1.37	7.0	1	10		

## Data Availability

The data presented in this study are available on request from the corresponding author.
